# Supplementary figures and images for: An analysis of the value-added of antibiogram subgroup stratification
Source: Ann Clin Microbiol Antimicrob. 2025 Apr 5;24:21. doi: 10.1186/s12941-025-00787-7 (PMC11972497; doi:10.1186/s12941-025-00787-7)

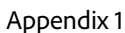

Supplement: Supplementary file 1 — Supplementary Material 1: Appendix 1 Heat map displaying differences in susceptibility percentages by individual organism/antimicrobial combinations for unit-specific (emergency department [ED], intensive care unit [ICU], transplant [TR] and units that are not ED, ICU nor TR [nIET]) all-specimens stratified antibiograms compared to the non-stratified hospital-wide all-specimens antibiogram. [file 12941_2025_787_MOESM1_ESM.pdf]

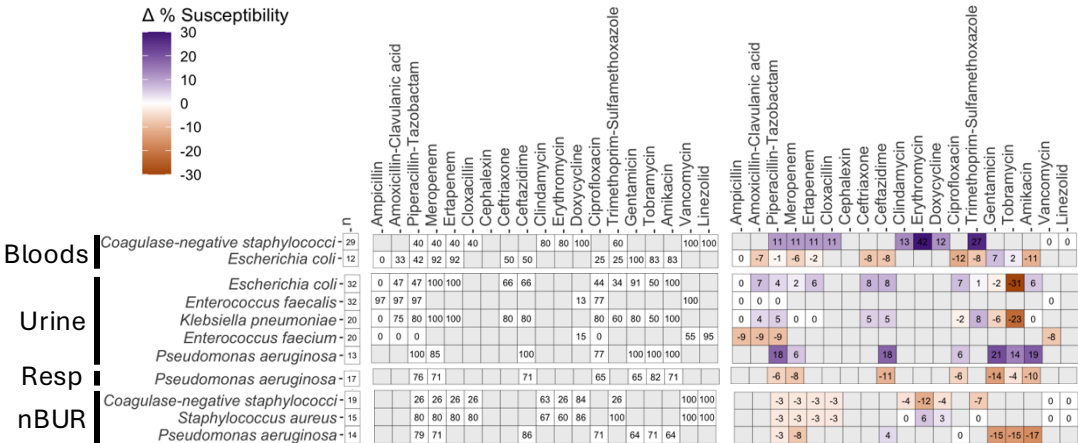

Supplement: Supplementary file 4 — Supplementary Material 4: Appendix 4 Heat map displaying differences in susceptibility percentages by individual organism/antimicrobial combinations for specimen-specific (blood, urine, respiratory [resp], and specimens that are not blood, urine nor resp [nBUR]) TR-only stratified antibiograms compared to the hospital-wide TR-only antibiogram. [file 12941_2025_787_MOESM4_ESM.pdf]
