# Supplementary material for: An analysis of the value-added of antibiogram subgroup stratification
Source: Ann Clin Microbiol Antimicrob. 2025 Apr 5;24:21. doi: 10.1186/s12941-025-00787-7 (PMC11972497; doi:10.1186/s12941-025-00787-7)
Supplement: Supplementary file 2 — Supplementary Material 2: Appendix 2 Heat map displaying differences in susceptibility percentages by individual organism/antimicrobial combinations for specimen-specific (blood, urine, respiratory [resp], and specimens that are not blood, urine nor resp [nBUR]) ED-only stratified antibiograms compared to the hospital-wide ED-only antibiogram. [file 12941_2025_787_MOESM2_ESM.pdf]

% Susceptibility

Specimen	Organism	n	Ampicillin	Amoxicillin-Clavulanic acid	Piperacillin-Tazobactam	Meropenem	Ertapenem	Cloxacillin	Cephalexin	Ceftriaxone	Ceftazidime	Clindamycin	Erythromycin	Doxycycline	Ciprofloxacin	Trimethoprim-Sulfamethoxazole	Gentamicin	Tobramycin	Amikacin	Vancomycin	Linezolid
Bloods	*Coagulase-negative staphylococci*	378			69	69	69	69				77	31	91		89				100	100
Bloods	*Escherichia coli*	131	47	73	79	100	100			79	79				79	73	95	92	97		
Bloods	*Staphylococcus aureus*	73			90	90	90	90				85	77	95		98				100	100
Bloods	*Klebsiella pneumoniae*	47	0	91	87	98	98			94	94				91	89	98	96	100		
Bloods	*Viridans group streptococci*	37								97										100	
Bloods	*Enterococcus faecalis*	23	100	100	100															100	
Bloods	*Pseudomonas aeruginosa*	17			88	100					88				88		100	100	100		
Bloods	*Proteus mirabilis*	17	80	100	100	100	100			100	100				100	88	94	100	100		
Bloods	*Group B streptococci*	14										86	71							100	
Bloods	*Streptococcus anginosus group*	13								100										100	
Bloods	*Group A streptococci*	13										77	77							100	
Urine	*Escherichia coli*	471	0	75	82	100	98			85	85			0	72	66	91	53	97		
Urine	*Enterococcus faecalis*	143	100	100	100									29	89					100	100
Urine	*Klebsiella pneumoniae*	112	0	88	88	100	100			91	91				90	81	96	33	100		
Urine	*Pseudomonas aeruginosa*	46			93	96					93				87		89	98	89		
Urine	*Proteus mirabilis*	46	25	93	93	100	100			93	93				83	85	93	100	67		
Urine	*Staphylococcus aureus*	39			85	85	85	85	85					95		97				100	100
Urine	*Enterobacter cloacae*	26	0	0	0	100	96		0	0	0				73	81	100	88	100		
Urine	*Enterococcus faecium*	21	9	9	9									19	5					86	100
Urine	*Staphylococcus saprophyticu*	17																			
Urine	*Klebsiella oxytoca*	15	0	87	87	100	100			87	87				100	93	93	50	100		
Urine	*Klebsiella aerogenes*	15	0	0	0	100	100		0	0	0				87	93	100	100	100		
Resp	*Pseudomonas aeruginosa*	14			86	85					86				86		71	93	85		
nBUR	*Staphylococcus aureus*	133			84	84	84	84				81	69	91		99				100	100
nBUR	*Pseudomonas aeruginosa*	42			98	98					98				83		95	100	98		
nBUR	*Escherichia coli*	18	0	61	61	100	100			78	78				67	61	94	75	100		
nBUR	*Coagulase-negative staphylococci*	18			56	56	56	56				78	56	89		67				100	100

Δ % Susceptibility

Δ % Susceptibility legend: 30, 20, 10, 0, -10, -20, -30

Specimen	Organism	n	Ampicillin	Amoxicillin-Clavulanic acid	Piperacillin-Tazobactam	Meropenem	Ertapenem	Cloxacillin	Cephalexin	Ceftriaxone	Ceftazidime	Clindamycin	Erythromycin	Doxycycline	Ciprofloxacin	Trimethoprim-Sulfamethoxazole	Gentamicin	Tobramycin	Amikacin	Vancomycin	Linezolid
Bloods	*Coagulase-negative staphylococci*	378			4	4	4	4				-3	-11	2		9				0	0
Bloods	*Escherichia coli*	131	23	0	-2	0	1			-4	-4				5	5	3	9	-1		
Bloods	*Staphylococcus aureus*	73			5	5	5	5				6	9	3		-1				0	0
Bloods	*Klebsiella pneumoniae*	47	0	0	-3	-1	0			0	0				0	4	1	5	0		
Bloods	*Viridans group streptococci*	37								-3										0	
Bloods	*Enterococcus faecalis*	23	1	1	0															0	
Bloods	*Pseudomonas aeruginosa*	17			-8	5					-7				2		9	2	6		
Bloods	*Proteus mirabilis*	17	13	7	5	0	0			5	5				17	7	3	6	6		
Bloods	*Group B streptococci*	14										5	9							0	
Bloods	*Streptococcus anginosus group*	13								0										0	
Bloods	*Group A streptococci*	13										8	10							0	
Urine	*Escherichia coli*	471	-24	2	1	0	-1			2	2				-2	-2	-1	-30	-1		
Urine	*Enterococcus faecalis*	143	1	1	0															0	
Urine	*Klebsiella pneumoniae*	112	0	-3	-2	1	2			-3	-3				-1	-4	-1	-58	0		
Urine	*Pseudomonas aeruginosa*	46			-3	1					-2				1		-2	0	-5		
Urine	*Proteus mirabilis*	46	-42	0	-2	0	0			-2	-2				0	4	2	6	-27		
Urine	*Staphylococcus aureus*	39			0	0	0	0						3		-2				0	0
Urine	*Enterobacter cloacae*	26	0	0	-2	0	-2			0	0				-8	-5	0	-5	0		
Urine	*Enterococcus faecium*	21	4	4	4															8	
Urine	*Staphylococcus saprophyticu*	17																			
Urine	*Klebsiella oxytoca*	15	0	-1	-1	0	0			-1	-1				0	5	-3	-42	0		
Urine	*Klebsiella aerogenes*	15	0	0	-5	0	0			0	0				-2	-1	0	0	0		
Resp	*Pseudomonas aeruginosa*	14			-10	-10					-9				0		-20	-5	-9		
nBUR	*Staphylococcus aureus*	133			-1	-1	-1	-1				2	1	-1		0				0	0
nBUR	*Pseudomonas aeruginosa*	42			2	3					3				-3		4	2	4		
nBUR	*Escherichia coli*	18	-24	-12	-20	0	1			-5	-5				-7	-7	2	-8	2		
nBUR	*Coagulase-negative staphylococci*	18			-9	-9	-9	-9				-2	14	0		-13				0	0

Appendix 2
