# Supplementary material for: An analysis of the value-added of antibiogram subgroup stratification
Source: Ann Clin Microbiol Antimicrob. 2025 Apr 5;24:21. doi: 10.1186/s12941-025-00787-7 (PMC11972497; doi:10.1186/s12941-025-00787-7)
Supplement: Supplementary file 3 — Supplementary Material 3: Appendix 3 Heat map displaying differences in susceptibility percentages by individual organism/antimicrobial combinations for specimen-specific (blood, urine, respiratory [resp], and specimens that are not blood, urine nor resp [nBUR]) ICU-only stratified antibiograms compared to the hospital-wide ICU-only antibiogram. [file 12941_2025_787_MOESM3_ESM.pdf]

% Susceptibility

Source	Organism	n	Ampicillin	Amoxicillin-Clavulanic acid	Piperacillin-Tazobactam	Meropenem	Ertapenem	Cloxacillin	Cephalexin	Ceftriaxone	Ceftazidime	Clindamycin	Erythromycin	Doxycycline	Ciprofloxacin	Trimethoprim-Sulfamethoxazole	Gentamicin	Tobramycin	Amikacin	Vancomycin	Linezolid
Bloods	*Coagulase-negative staphylococci*	251			20	20	20	20				50	35	95		50				100	100
	Staphylococcus aureus	67			88	88	88	88				75	66	94		100				100	100
	Enterococcus faecalis	41	100	100	100															100	
	Klebsiella pneumoniae	25	0	84	84	96	96			84	84				88	88	100	96	100		
	Enterococcus faecium	24	21	21	21															79	
	Escherichia coli	19	38	53	58	100	100			58	58				58	58	84	68	84		
	Streptococcus anginosus group	12								100										100	
	Viridans group streptococci	11								91										100	
Urine	*Escherichia coli*	24	0	62	62	96	67		0	71	71				71	67	88	0	100		
	Enterococcus faecalis	23	100	100	100									35	96					100	
	Pseudomonas aeruginosa	14			79	93					79				71		79	93	93		
Resp	*Staphylococcus aureus*	152			88	88	88	88				83	75	94		98				100	100
	Pseudomonas aeruginosa	81			72	75					72				73		85	95	84		
	Klebsiella pneumoniae	41	0	80	80	98	88			80	80				90	90	95	75	88		
	Serratia marcescens	23	0	0	0	100	100			0	0				91	100	100	96	100		
	Enterobacter cloacae	22	0	0	0	95	91			0	0				100	86	100	100	100		
	Haemophilus influenzae	21	76																		
	Stenotrophomonas maltophilia	20														95					
	Klebsiella oxytoca	19	0	84	84	100	100			84	84				100	100	100	100	100		
	Klebsiella aerogenes	19	0	0	0	100	100			0	0				100	100	100	100	100		
	Escherichia coli	19	0	42	47	100	100			58	58				68	58	89	33	100		
	Citrobacter koseri	14	0	0	0	100	100			0	0				100	100	100	100	100		
nBUR	*Coagulase-negative staphylococci*	25			24	24	24	24				52	44	88		40				100	100
	Staphylococcus aureus	22			86	86	86	86				72	55	96		100				100	100
	Escherichia coli	16	15	50	50	100	100			69	69				75	62	88	67	89		
	Pseudomonas aeruginosa	14			71	57					64				71		86	93	86		
	Enterococcus faecium	11	9	9	9															64	

Δ % Susceptibility

Δ % Susceptibility (colour scale): 30, 20, 10, 0, -10, -20, -30

Source	Organism	n	Ampicillin	Amoxicillin-Clavulanic acid	Piperacillin-Tazobactam	Meropenem	Ertapenem	Cloxacillin	Cephalexin	Ceftriaxone	Ceftazidime	Clindamycin	Erythromycin	Doxycycline	Ciprofloxacin	Trimethoprim-Sulfamethoxazole	Gentamicin	Tobramycin	Amikacin	Vancomycin	Linezolid
Bloods	*Coagulase-negative staphylococci*	251			0	0	0	0				-2	-2	4		2				0	0
	Staphylococcus aureus	67			-1	-1	-1	-1				-5	-8	0		1				0	0
	Enterococcus faecalis	41	0	0	0															0	
	Klebsiella pneumoniae	25	0	3	3	-2	0			3	3				0	0	2	0	0		
	Enterococcus faecium	24	-1	-1	-1															4	
	Escherichia coli	19	16	-3	-1	1	3			-8	-8				-10	-8	-4	9	-4		
	Streptococcus anginosus group	12								0										0	
	Viridans group streptococci	11								-1										0	
Urine	*Escherichia coli*	24	-22	6	3	-3	-30			5	5				3	1	0	-59	12		
	Enterococcus faecalis	23	0	0	0															0	
	Pseudomonas aeruginosa	14			5	17					6				-4		-7	-2	6		
Resp	*Staphylococcus aureus*	152			-1	-1	-1	-1				3	1	0		-1				0	0
	Pseudomonas aeruginosa	81			-2	-1					-1				-2		-1	0	-3		
	Klebsiella pneumoniae	41	0	-1	-1	0	-8			-1	-1				2	2	-3	-21	-12		
	Serratia marcescens	23	0	0	0	0	0			0	0				-1	0	0	0	0		
	Enterobacter cloacae	22	0	0	0	1	1			0	0				0	-1	0	0	0		
	Haemophilus influenzae	21	3																		
	Stenotrophomonas maltophilia	20														4					
	Klebsiella oxytoca	19	0	0	0	0	0			0	0				4	8	4	14	0		
	Klebsiella aerogenes	19	0	0	0	0	0			0	0				5	0	0	0	0		
	Escherichia coli	19	-22	-14	-12	1	3			-8	-8				0	-8	1	-26	12		
	Citrobacter koseri	14	0	0	-6	0	0			0	0				0	0	0	0	0		
nBUR	*Coagulase-negative staphylococci*	25			4	4	4	4				0	7	-3		-8				0	0
	Staphylococcus aureus	22			-3	-3	-3	-3				-8	-19	2		1				0	0
	Escherichia coli	16	-7	-6	-9	1	3			3	3				7	-4	0	8	1		
	Pseudomonas aeruginosa	14			-3	-19					-9				-4		0	-2	-1		
	Enterococcus faecium	11	-13	-13	-13															-11	

Appendix 3
