# Supplementary material for: An analysis of the value-added of antibiogram subgroup stratification
Source: Ann Clin Microbiol Antimicrob. 2025 Apr 5;24:21. doi: 10.1186/s12941-025-00787-7 (PMC11972497; doi:10.1186/s12941-025-00787-7)
Supplement: Supplementary file 5 — Supplementary Material 5: Appendix 5 Heat map displaying differences in susceptibility percentages by individual organism/antimicrobial combinations for specimen-specific (blood, urine, respiratory [resp], and specimens that are not blood, urine nor resp [nBUR]) nEIT-only stratified antibiograms compared to the hospital-wide nEIT-only antibiogram. [file 12941_2025_787_MOESM5_ESM.pdf]

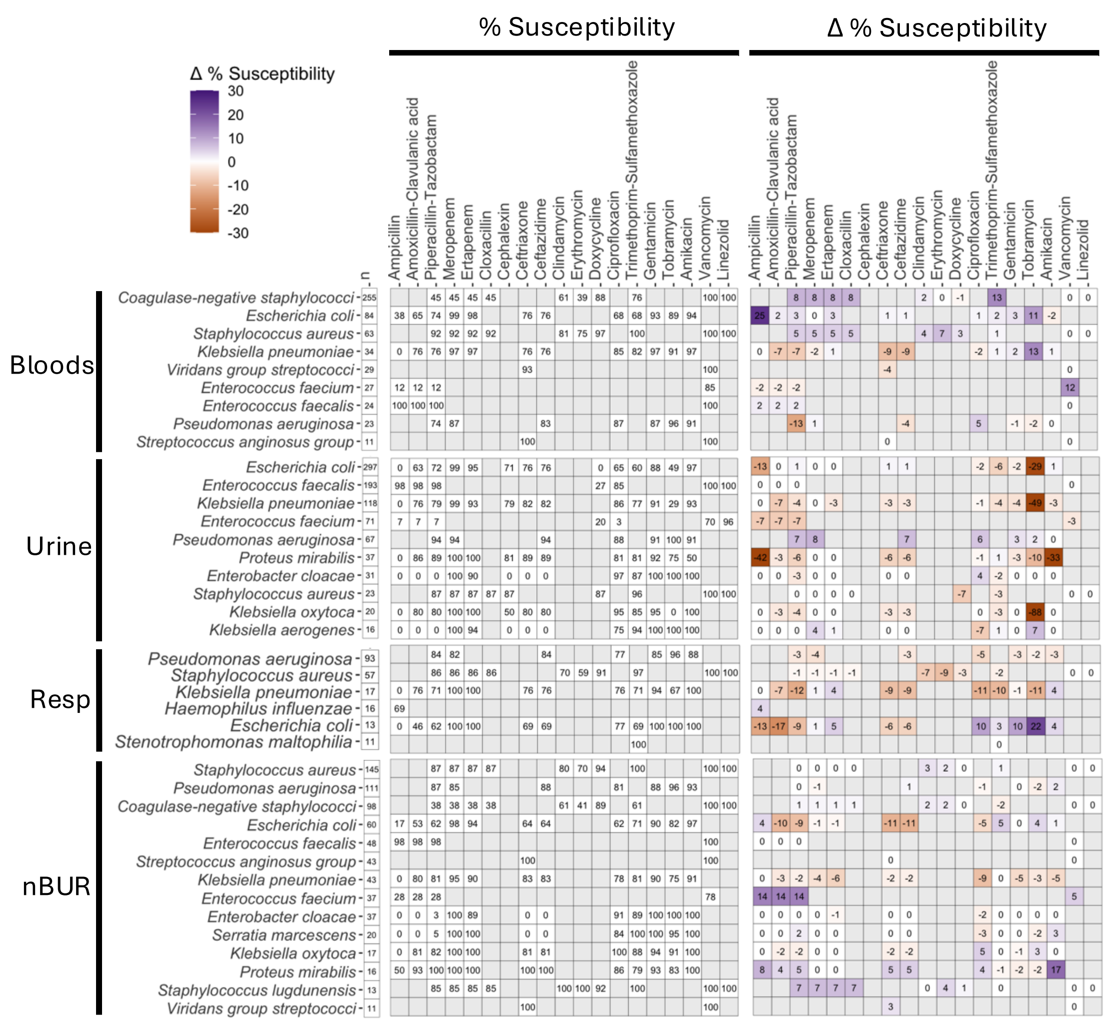
% Susceptibility
Δ % Susceptibility
Δ % Susceptibility
30
20
10
0
-10
-20
-30
n
Ampicillin
Amoxicillin-Clavulanic acid
Piperacillin-Tazobactam
Meropenem
Ertapenem
Cloxacillin
Cephalexin
Ceftriaxone
Ceftazidime
Clindamycin
Erythromycin
Doxycycline
Ciprofloxacin
Trimethoprim-Sulfamethoxazole
Gentamicin
Tobramycin
Amikacin
Vancomycin
Linezolid
Bloods
Coagulase-negative staphylococci
Escherichia coli
Staphylococcus aureus
Klebsiella pneumoniae
Viridans group streptococci
Enterococcus faecium
Enterococcus faecalis
Pseudomonas aeruginosa
Streptococcus anginosus group
Urine
Escherichia coli
Enterococcus faecalis
Klebsiella pneumoniae
Enterococcus faecium
Pseudomonas aeruginosa
Proteus mirabilis
Enterobacter cloacae
Staphylococcus aureus
Klebsiella oxytoca
Klebsiella aerogenes
Resp
Pseudomonas aeruginosa
Staphylococcus aureus
Klebsiella pneumoniae
Haemophilus influenzae
Escherichia coli
Stenotrophomonas maltophilia
nBUR
Staphylococcus aureus
Pseudomonas aeruginosa
Coagulase-negative staphylococci
Escherichia coli
Enterococcus faecalis
Streptococcus anginosus group
Klebsiella pneumoniae
Enterococcus faecium
Enterobacter cloacae
Serratia marcescens
Klebsiella oxytoca
Proteus mirabilis
Staphylococcus lugdunensis
Viridans group streptococci

Appendix 5
